# Genetic polymorphism of merozoite surface protein 2 and prevalence of K76T *pfcrt *mutation in *Plasmodium falciparum *field isolates from Congolese children with asymptomatic infections

**DOI:** 10.1186/1475-2875-11-105

**Published:** 2012-04-01

**Authors:** Felix Koukouikila-Koussounda, Vladimir Malonga, Pembe Issamou Mayengue, Mathieu Ndounga, Christevy Jeannhey Vouvoungui, Francine Ntoumi

**Affiliations:** 1 Congolese Foundation for Medical Research, Brazzaville, Republic of Congo; 2 Faculty of Health Sciences, Marien Ngouabi University, Brazzaville, Republic of Congo; 3 Centre de Recherche sur les Ressources Végétales, Brazzaville, Republic of Congo; 4 Unité de Recherche sur le Paludisme, Hôpital de Base de Makélékélé, Brazzaville, Republic of Congo; 5 Institute for Tropical Medicine, University of Tübingen, Tübingen, Germany

**Keywords:** *Plasmodium falciparum*, Asymptomatic infection, Multiplicity of infection, *msp2*, *pfcrt*, Brazzaville, Republic of Congo

## Abstract

**Background:**

In order to prepare the field site for future interventions, the prevalence of asymptomatic *Plasmodium falciparum *infection was evaluated in a cohort of children living in Brazzaville. *Plasmodium falciparum *merozoite surface protein 2 gene (*msp*2) was used to characterize the genetic diversity and the multiplicity of infection. The prevalence of mutant *P. falciparum *chloroquine resistance transporter (*pfcrt*) allele in isolates was also determined.

**Methods:**

Between April and June 2010, 313 children below 10 years of age enrolled in the cohort for malaria surveillance were screened for *P. falciparum *infection using microscopy and polymerase chain reaction (PCR). The children were selected on the basis of being asymptomatic. *Plasmodium falciparum msp2 *gene was genotyped by allele-specific nested PCR and the *pfcrt *K76T mutation was detected using nested PCR followed by restriction endonuclease digestion.

**Results:**

The prevalence of asymptomatic *P. falciparum *infections was 8.6% and 16% by microscopy and by PCR respectively. Allele typing of the *msp2 *gene detected 55% and 45% of 3D7 and FC27 allelic families respectively. The overall multiplicity of infections (MOI) was 1.3. A positive correlation between parasite density and multiplicity of infection was found. The prevalence of the mutant *pfcrt *allele (T76) in the isolates was 92%.

**Conclusion:**

This is the first molecular characterization of *P. falciparum *field isolates in Congolese children, four years after changing the malaria treatment policy from chloroquine (CQ) to artemisinin-based combination therapy (ACT). The low prevalence of asymptomatic infections and MOI is discussed in the light of similar studies conducted in Central Africa.

## Background

Despite significant reduction in malaria-related morbidity and mortality in the recent past, malaria remains endemic in the tropics and sub-tropics including sub-Saharan Africa. About 225 million clinical cases and 781,000 deaths were reported worldwide in 2009, whereby almost 90% occurred in sub-Saharan Africa [1]. Current malaria control strategies include the use of insecticide-treated bed nets (ITNs), indoor residual spraying of insecticide, intermittent preventive treatment to young children and pregnant women, and early parasitological diagnosis and treatment of clinical cases using artemisinin-based combination therapy (ACT) [2-4]. Deployment of these strategies has had significant impact on malaria in many endemic areas.

Although the impact of current malaria control strategies is encouraging, much more remains to be done to attain elimination. In the meantime, malaria vaccine is considered as an additional necessary arsenal. However, genetic diversity in *Plasmodium falciparum *is a major limitation for the successful development of an effective malaria vaccine, as it influences the level and efficacy of acquired protective immunity to malaria. Therefore, screening of the genetic diversity of malaria parasite populations in different endemic settings is an important step towards the development and/or the evaluation of malaria vaccines [5].

Genotyping of merozoite surface protein-2 (*msp2*) is the commonly used method to study the genetic diversity of *P. falciparum*. This gene presents polymorphisms in the number of repeats and sequence type [6]. The analysis of *msp2 *alleles involves two major allelic families, FC27 and 3D7. The *msp2 *gene is also the basis of determining the multiplicity of infections (MOI) in infected individuals, which is defined as the number of distinguishable *P. falciparum *clones *per *infected individual. MOI is a good indicator of acquired immunity or premunition of the populations living in endemic areas and is also correlated to the transmission intensity [7,8]. It has been demonstrated that MOI could be influenced by parasite density and age [9], and haemoglobin (Hb) type carriage [10].

Makélékélé is an administrative division in the south-eastern part of Brazzaville (Republic of Congo), and has been well characterized as a highly endemic area with perennial malaria transmission [11,12]. However, there is a paucity of information on the genetic diversity of *P. falciparum *populations. Following the replacement of chloroquine (CQ) with ACT (artesunate-amodiaquine and artemether-lumefantrine as first and second line treatment respectively) in 2006 for the treatment of uncomplicated malaria in the Republic of Congo [13], CQ drug pressure has since been reduced. In Malawi, absence of CQ pressure was followed by the re-emergence of CQ-susceptible malaria, based on genotyping of CQ resistance biomarker, *P. falciparum *chloroquine resistance transporter gene (*pfcrt*) [14-16]. In Congo Brazzaville, molecular analysis of the parasite population, including the CQ resistance *pfct *K76T mutation has not been investigated since the change of anti-malarial treatment policy in 2006. Therefore, the present study was conducted in the semi-urban area of Makélékélé to determine (i) the prevalence and the multiplicity of *P. falciparum *asymptomatic infection; (ii) the genetic diversity of the *msp2 *gene; and (iii) the prevalence of *pfcrt *K76T mutation in *P. falciparum *isolates.

## Methods

### Study site

The study conducted under the auspices of the Central Africa Network on Tuberculosis, HIV/AIDS and Malaria (CANTAM) project, was carried out in three districts of Makélékélé health division: Ngoko, Kinsana and Mbouono. These semi-urban divisions with about 6,000 inhabitants are located in the southern part of Brazzaville along the Congo River where malaria is transmitted throughout the year [12]. *Plasmodium falciparum *is the main plasmodial species and *Anopheles gambiae *the main mosquito vector.

### Study population

Children aged one to nine years and permanent residents of the study area were enrolled in a cohort for malaria surveillance study (Ndounga *et al*, unpublished). From October 2009 to June 2010, a demographic survey was conducted and 313 children under 10 years were enrolled. Malaria incidence is 0.9 malaria episode/year/child. Inclusion criteria were absence of clinical malaria in the last two weeks and at least one week after enrolment, and with an axillary temperature of < 37.5°C. Recruitment was done from April to June 2010, and informed consent was obtained from parents or guardians. Thick and thin blood smears were made from each child. About 4 ml of whole blood were also collected in EDTA tube for haemoglobin concentration measurement and DNA extraction. This study was approved by the Institutional Ethics Committee for Research on Heath Sciences of the Republic of Congo.

### Microscopic examination

Thick and thin blood films were stained with 10% Giemsa for 15 min and read by two independent competent microscopists to determine malaria species and the parasite density. Asexual parasites were counted against 200 leucocytes and expressed as the number of asexual parasites/μl of blood, assuming the leucocyte count of 8,000/μl of blood.

### Genomic DNA extraction

Genomic DNA was extracted from 200 μl of whole blood sample using the QIAmp DNA Blood Mini kit (QIAGEN Gmbh, Hilden, Germany) following the manufacturer's instruction. DNA was recovered in 200 μl of elution buffer provided in the kit and stored at -20°C until use.

### *msp*2 genotyping of *plasmodium falciparum *isolates

To characterize parasite infection, *P. falciparum *genotyping was performed using a nested PCR for *msp2 *central region, which comprises repeats of varying lengths. The FC27 and 3D7 allele families were analysed. The specific oligonucleotide primers and PCR conditions were as previously described by Ntoumi *et al *[17].

### *Pfcrt *K76T PCR-typing

The nested PCR followed by restriction enzyme digestion PCR reactions were used to genotype the Lysine to Threonine mutation in codon 76 of *pfcrt *as described by Mayengue *et al *[18], Djimde *et al *[19] and Mayor *et al *[20]. All isolates successfully amplified using *msp2 *PCR were assayed with the *pfcrt *PCR.

### Sickle cell trait

β-globin genotypes HbAA, HbAS and HbSS were determined using allele specific PCR method as described by Wu *et al *[21].

### Data analysis

The prevalence of FC27 and 3D7 alleles was determined as the presence of PCR products for each type in the total number of amplified bands for the corresponding locus. The MOI was determined as the number of different *msp2 *genotypes per isolate, and the mean MOI was calculated as the total number of detected *Plasmodium falciparum msp2 *genotypes/total number of infected children [18]. The *msp2 *allelic frequency represents the ratio of one specific allele out of the total number of alleles identified in the isolates. The polyclonality (percentage of isolates with more than one amplified PCR fragment) was estimated for each group. Similarly, proportions of wild and mutant *pfcrt *alleles were calculated as the ratio of each allele type out of the total of *P. falciparum *positive samples. Statistical analysis was done using XLSTAT software (Version 2011.2.08). The chi-square test was used to compare quantitative variables such as parasite prevalence and MOI between different groups. The Spearman's rank correlation coefficient was calculated to assess association between MOI and parasite density. Differences were considered statistically significant at *P *values < 0.05.

## Results

### Baseline demographic data and parasitological indexes

A total of 313 children aged from one to nine years (mean age: 4.4 ± 2.5 years) were enrolled in the study. Sickle cell trait was present in 58 (18.5%) of them. The haemoglobin levels were distributed into 3 categories: normal ≥ 12.0 g/dl (23.3% of children), moderate anemia 11.9 g/dl-7.0 g/dl (72.2%) and severe anemia < 7 g/dl (1.3%).

The prevalence of asymptomatic parasitaemia detected microscopically was 8.6%, *P. falciparum *being the only infecting specie. The parasite density ranged from 62 to 16,090 parasites/μl and a geometric mean density of 3,421 parasites/μl. Through PCR amplification of *msp2 *gene, *P. falciparum *infection was detected in 16% of the isolates (Table 1).

**Table 1:** Demographic data and parasitological indexes of the study population

Parameters	Values
Mean age (year)	4.4 ± 2.5
Age range	1-9
Sex ratio (M/F)	1.1 (168/146)
Mean axillary temperature (°C)	36.5 ± 0.3
Axillary temperature range (°C)	36-37.4
Mean hematocrit (g/dl)	10.0
Hb ≥ 12.0 g/dl n(%)	73 (23.3%)
Hb 7.0 g/dl -11.9 g/dl n(%)	226 (72.2%)
Hb < 7.0 g/dl n(%)	4 (1.3%)
Hb not determined	10 (3.2%)
HbAA, n(%)	255 (81.5%)
HbAS, n(%)	58 (18.5%)
HbSS, n(%)	0
*P. falciparum *prevalence, n(%)	
Microscopy	27 (8.6%)
PCR	51 (16.0%)
Geometric mean parasite density (p/μl)	3,421
Parasite density range (p/μl)	62-16,090
Mean MOI	1.3
MOI-range	1-3
*pfcrt *K76T prevalence n(%)*	
Wild type	4 (8%)
Mutant type	47 (92%)
Number of individuals	313

* Prevalence determined among PCR positive samples

The prevalence of infection (by microscopy and PCR) varied according to the age of children (P < 0.05), being highest in the seven to nine years age group (14 and 26% respectively), followed by four to six years (11 and 17%), and one to three years age group (3.5 and 7%).

### Merozoite surface protein 2 genetic diversity

**Figure 1:**
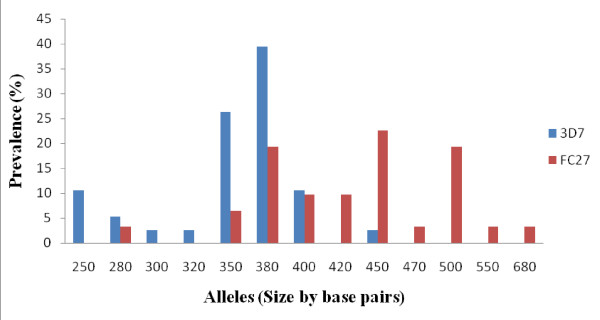
**Distribution of *P. falciparum *3D7 and FC27 *msp2 *alleles**.

Alleles were determined by size and family type. Allele typing showed the highly polymorphic nature of *P. falciparum *isolates. Both 3D7 and FC27 families were identified with 8 and 10 different alleles respectively (Figure 1). The proportion of isolates having only 3D7 and FC27 alleles was 23/51 (45%) and 18/51 (35%) respectively. Both allelic families were found in 7/51(14%) of the isolates. Six per cent (6%) of the isolates were not identified to any of the 3D7 or FC27 allelic family despite being repeatedly amplified (Table 2). This might be due to a mutation in the annealing region of the primers.

**Table 2:** Genetic diversity of *P. falciparum msp*2 gene

	No. of samples	Prevalence (%)	Fragment size (bp)	Total No. of alleles^a^	Allelic frequency^b^
3D7	23	45	250-450	38	55
FC27	18	35	280-680	31	45
FC27 + 3D7	7	14			
Non identified	3	6			
Total	51	100		69	100

No. Number

^a ^Total number of alleles is the number of different *msp2 *alleles distinguished by the size and the family type (3D7 and FC27)

^b ^Allelic frequency is defined as the ratio of 3D7 or FC27 alleles out of the total number of *msp2 *alleles

### Multiplicity of *plasmodium falciparum *infection

In this study, 74% of the infections analysed consisted of a single *msp2 *genotype, 22% were double infection and 4% triple. The overall MOI was 1.3 and similar in all age groups. The proportion of samples with more than one clone was higher in both one to three and four to six years age groups (33.3 and 27.8% respectively) compared to the seven to nine years olds group (19%) (Table 3). However, the difference was not statistically significant.

**Table 3:** Comparison of the parasite prevalence, parasite density and the MOI in different age groups and haemoglobin (Hb) phenotypes

	Age groups			*P*	Hb type		*P*
				
	1-3	4-6	7-9		AA	AS	
	N = 137	N = 96	N = 80		N = 255	N = 58	
Parasite prevalence n(%)							
Microscopy	5 (3.5%)	11 (11%)	11(14%)	0.018	19 (7.5%)	8 (13.5%)	NS
PCR	12 (8.8%)	18 (17%)	21(26%)	0.002	40 (15.5%)	11 (19%)	NS
Geometric mean parasite density (p/μl)	1,246	3,442	3,024	NS	4,052	2,081	NS
Mean MOI	1.3	1.3	1.2	NS	1.3	1.4	NS
Proportion of multiple infections*	4/12(33.3%)	5/18(27.8%)	4/21(19%)	NS	8/40(19%)	3/11(27%)	NS

* Proportion of multiple infections in PCR positive samples, NS non significant

**Figure 2:**
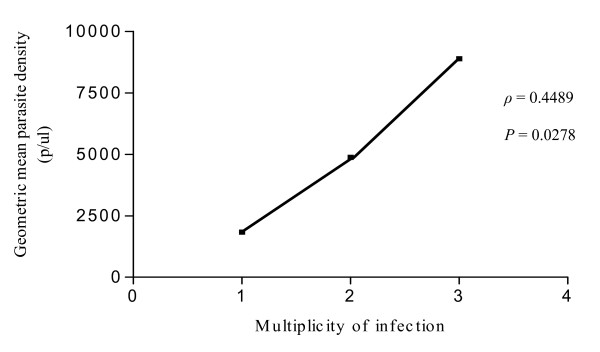
**Correlation of *msp2 *multiplicity of infection and mean *Plasmodium falciparum *parasite density Spearman rank coefficient = 0.4489, *P *= 0.0278**.

The haemoglobin phenotype (AA or AS) had no influence on MOI. The prevalence of multiple infections in AA group was 19% compared with 27% in AS group, and this difference was not significant (Table 3). Nonetheless, a significant positive association was found between *P. falciparum *parasite density and MOI (Spearman rank coefficient = 0.4489, *P *= 0.0278) (Figure 2).

### Prevalence of *pfcrt *K76T mutation

Genotyping of the *pfcrt *gene showed that 47 isolates out of 51 (92%) produced the expected 134 bp amplicon, suggesting the presence of T76 mutation. Four isolates (8%) with the wild type were found (Table 1).

## Discussion

In the Republic of Congo, less attention had been put on the investigation of the genetic diversity of *P. falciparum *among asymptomatic parasite carriers. Besides, this analysis is necessary to develop strategies for malaria control including the design of malaria vaccines interventions. The current study is the first of its kind to evaluate genetic diversity of *P. falciparum *isolates circulating among children younger than 10 years old, and to present the molecular analysis of *pfcrt *gene since the replacement of chloroquine with ACT as the first line anti-malarial drug in Congo Brazzaville.

The prevalence of *P. falciparum *carriage of 8.6% (microscopically) and 16% (by PCR) is low compared to those reported previously from other malaria endemic areas [9,10,22]. However, these findings are in line with the results of a recent study conducted in Gabon that showed a relatively low prevalence of asymptomatic *P. falciparum *infection of 5% by microscopy among young children [23]. In the recent past, a large number of ITNs were available to children under five years of age and to pregnant women in the Republic of Congo. The observed low prevalence could be attributed to the wide use of the ITNs. Further investigations involving more children and adults would be of great importance to confirm this finding.

Allele-specific genotyping of *msp2 *has shown that *P. falcuparum *population in this part of the Republic of Congo have a high genetic diversity. Dispite the fact that a high proportion of isolates (74%) carried one genotype in comparison to those having two and three genotypes, different types of both 3D7 and FC27 alleles were found. The proportion of 3D7 alleles (55%) was slightly high compared with that of FC27 (45%). This is similar to findings in Papua New Guinea [22].

The MOI (number of concurrent *msp2 *genotypes in individual infections) ranged from 1 to 3. The mean MOI of 1.3 is relatively low compared to those reported in Gabon [24], Senegal [9,25], and Ghana [26] in asymptomatic cases. This might be explained by the fact that diversity of *P. falciparum *population differs according to the geographic location, and the level of transmission [27,28]. In the same study area [29], the MOI in isolates from symptomatic children was 2.2. The interpretation of this increase in the mean number of parasite genotype/infected child could be the presence of the uncontrolled strain causing the symptoms.

Age is one of the suggested important factors involved in the acquisition of immunity against *P. falciparum *and influences MOI [9]. On the contrary, these results show that age has no effect on MOI. The absence of influence of age on MOI has also been previously reported in the same area among *P. falciparum *symptomatic children by Pembe *et al *[29].

Previous studies on association between MOI and parasite densities showed that high parasite densities increase the probability of detecting concurent clones in an individual [30,31]. Expectedly, a positive correlation between MOI and parasite density was found in the present study.

The impact of sickle cell trait on MOI in this study is different from findings in Gabon by Ntoumi *et al *[10], in which, a correlation between sickle cell trait and MOI was found. However, this observation corroborates reports from Senegale by Konate *et al *[32] and Vafa *et al *[9] whereby no correlation was found. These discrepancies can be explained by differences in age ranges of the study population and the number of individuals with sickle cell trait enrolled.

The prevalence of *pfcrt *K76T was also assessed, the mutation associated with CQ resistance. It was anticipated that the sensitivity of the parasite to CQ may have been restored several years after its withdrawal as was the case in Malawi [16]. The prevalence of *Pfcrt *K76T, an important molecular marker surveillance tool that can contribute to the evaluation of local parasite strains sensitivity to CQ in addition to *in vitro *studies [33], was 98% in Congo before withdrawal of CQ [18]. Almost five years later, this study shows that the prevalence of this mutation remains high, 92%. This observation is similar to a recent report by Efunshile *et al *in Nigeria [34]. Therefore, CQ cannot be re-introduced in Congo now despite its many advantages over other anti-malarials.

## Conclusion

In summary, these findings shows that multiplicity of infection which is low in Congolese children with asymptomatic *P. falciparum *infection increases with parasite density. The prevalence of the mutant *pfcrt *allele remains extremely high in the study area. Further investigations with larger sample size in different zones of the country would be needed to confirm these observations.

## Abbreviations

*msp*2: merozoite surface protein 2 gene; *pfcrt: Plasmodium falciparum *chloroquine resistance transporter; PCR: Polymerase chaine reaction; MOI: Multiplicity of infection; ITN: Insecticide-treated bed net; ACT: Artemisinin-based combination therapy; CQ: Chloroquine; Hb: Heamoglobin; UV: Ultraviolet; bp: Base pair; dNTP: Deoxynucleoside triphosphate; MgCl2: Magnesium dichloride.

## Competing interests

The authors declare that they have no competing interests.

## Authors' contributions

FKK performed DNA extraction, molecular genetic studies, data analysis, and drafted the manuscript. VM and PIM participated in molecular genetic studies, CJV participated in the data analysis. MN designed and supervised the field study. FN supervised the different steps of the work. All authors contributed to the final manuscript.
